# Passive Smoking and Risk of Type 2 Diabetes: A Meta-Analysis of Prospective Cohort Studies

**DOI:** 10.1371/journal.pone.0069915

**Published:** 2013-07-26

**Authors:** Ying Wang, Jie Ji, Yu-jian Liu, Xuan Deng, Qi-qiang He

**Affiliations:** 1 School of Public Health, Wuhan University, Wuhan, P. R. China; 2 School of Public Health, Tongji Medical College, Huazhong University of Science and Technology, Wuhan, P. R. China; 3 Global Health Institute, Wuhan University, Wuhan, P. R. China; Universidad Peruana de Ciencias Aplicadas (UPC), Peru

## Abstract

**Backgrounds/Objective:**

The prevalence of diabetes is increasing rapidly all over the world. However, studies on passive smoking and type 2 diabetes have not been systematically assessed. Therefore, we conducted a meta-analysis to explore whether an association exists between passive smoking and risk of type 2 diabetes.

**Methods:**

We searched PubMed, EMBASE, Cochrane library and Web of Science up to April 9^th^, 2013, to identify prospective cohort studies that assessed passive smoking and risk of type 2 diabetes. The fixed-effect model was used to calculate the overall relative risk (RR).

**Result:**

4 prospective cohort studies were included for analysis, with a total of 112,351 participants involved. The pooled RR was 1.28 (95% confidence interval (CI) 1.14 to 1.44) comparing those who were exposed to passive smoking with those who were not. Subgroup, sensitivity analysis and publication bias test suggested the overall result of this analysis was robust.

**Conclusions:**

Passive smoking is associated with a significantly increased risk of type 2 diabetes. Further well-designed studies are warranted to confirm this association.

## Introduction

The prevalence of diabetes is increasing rapidly all over the world, and it is estimated that 439 million adults will be affected by diabetes by 2030 [1]. Type 2 diabetes(T2DM), which is characterized by reduced insulin sensitivity and relative insulin deficiency [2], consists of over 95% of diabetes worldwide [3]. Therefore, identification of risk factors of T2DM is of significant importance to the primary prevention of this disease.

A recent study reported that 40% of children, 33% of male non-smokers and 35% of female non-smokers were exposed to passive smoking worldwide [4]. It has been shown that passive smoking can cause disease, disability, and death [5]. However, the association between passive smoking and T2DM risk has not been summarized. Therefore, we performed this meta-analysis to systematically assess the association between passive smoking and risk of T2DM based on prospective cohort studies.

## Methods

### Data sources and searches

We conducted this meta-analysis according to the Meta-Analysis of Observational Studies in Epidemiology guidelines [6]. We performed a systematic search of PubMed, EMBASE, the Cochrane library and Web of Science up to April 9^th^, 2013 to identify relevant prospective cohort studies regarding the association between passive smoking and risk of T2DM. We also searched the reference lists of all retrieved articles to identify any additional literatures. However, we did not search the gray literature. There was no language restriction.

The search terms were (Diabetes mellitus, type 2 OR Diabetes mellitus OR Prediabetic state OR impaired fasting glucose OR impaired glucose tolerance OR Metabolic syndrome OR Glucose intolerance OR Hyperglycemia OR Glucose metabolism disorders OR Insulin resistance OR Glucose) AND (Tobacco smoke pollution OR Passive smoking OR Air pollution, tobacco smoke OR Second-hand smoking OR Involuntary smoking) (Table 1).

**Table 1:** Search strategy for PubMed (up to April 9^th^, 2013).

Search strategy	Search terms
#1	Diabetes mellitus, type 2
#2	Diabetes mellitus
#3	Prediabetic state
#4	Impaired fasting glucose
#5	Impaired glucose tolerance
#6	Metabolic syndrome
#7	Glucose intolerance
#8	Hyperglycemia
#9	Glucose metabolism disorders
#10	Insulin resistance
#11	Glucose
#12 #1 AND #2 AND #3 AND #4 AND #5 AND #6 AND #7 AND #8 AND #9 AND #10 AND #11	
#13	Tobacco smoke pollution
#14	Passive smoking
#15	Air pollution, tobacco smoke
#16	Secondhand smoking
#17	Involuntary smoking
#18 #13 AND #14 AND #15 AND #16 AND #17	
#19 #12 AND #18	

### Study selection

We first screened the titles and abstracts of all the articles to identify the possible eligible studies, and then read the full articles to include eligible studies. Studies were included if they met the following criteria: had a prospective cohort design, the exposure was passive smoking, the outcome was T2DM, reported estimates of the odds ratio (OR) or relative risk (RR) or hazard ratio (HR) and its 95% confidence interval (CI) or reported data to calculate them. Only the latest study was included if there were duplicates or data were originated from the same study population. Review or studies that did not report available information were excluded.

### Data extraction and quality assessment

Data extraction was conducted by two independent reviewers; disagreements would be resolved by consensus. The reference groups were never smokers who were not exposed to passive smoking, except that in one study the reference group were“those currently exposed to passive smoke but did not actively smoke, irrespective of past smoke” [7], The corresponding risk estimates (including RRs, ORs and HRs) adjusted for the maximum number of confounding variables with corresponding 95%CIs were extracted. We also extracted the following data: name of the first author, publication year, study location, age of the participants, total number of patients and participants involved, percentage of female, information of exposed groups, confounding factors that were adjusted for in the analysis.

A 9-star system based on the Newcastle-Ottawa Scale (NOS) [8] was employed for quality assessment. 4, 2, 3 scores were respectively assigned for selection of study groups, comparability of study groups, assessment of outcomes and adequacy of follow-up. Studies with scores of 0–3, 4–6, 7–9 were considered as low, moderate and high quality, respectively.

### Data analysis

The RR was used as the common measure of association across studies. As HR was broadly equivalent to relative risk (RR) [9], [10], HRs were directly considered as RRs. ORs were transformed into RRs according to the formula RR = OR/[(1−P_0_)+(P_0_×OR)] where P_0_ stands for the incidence of T2DM in nonexposed group [11]. In addition, the Miettinen test-based approach was used to calculate the variance of lnRR (variance lnRR = variance lnOR×[lnRR/lnOR]) [12]. Heterogeneity across studies was assessed using the Cochrane *Q* statistic (significance level at *P*<0.10) and the *I^2^* statistic [13], [14]. The heterogeneity was considered statistically insignificant if *P*>0.10 and *I^2^*≤50%, then the Mantel-Haenszel fixed-effect model was used to calculate pooled RR among studies. Otherwise, the DerSimonian and Laird [15] random-effect model was used to combine the results. Sensitivity analysis was performed to detect the effects of individual study on the pooled result by omitting one study in each turn. We conducted the subgroup analyses according to study location, percentage of female, study quality, and confounding factors being adjusted for to examine the cause of potential heterogeneity.

Potential publication bias was detected by both Begg's test [16] and Egger's test [17]. In order to further assess the possible effect of publication bias on the pooled RR, the nonparametric trim and fill method [18] was used. This method considers the possibility of hypothetical “missing” studies that might exist, then imputes their RRs, and recalculates a pooled RR which incorporates the hypothetical missing studies as though they actually exist. STATA version 11.0 (Stata Corporation, College Station, TX) was employed to conduct all data analysis.

## Results

### Literature search

**Figure 1:**
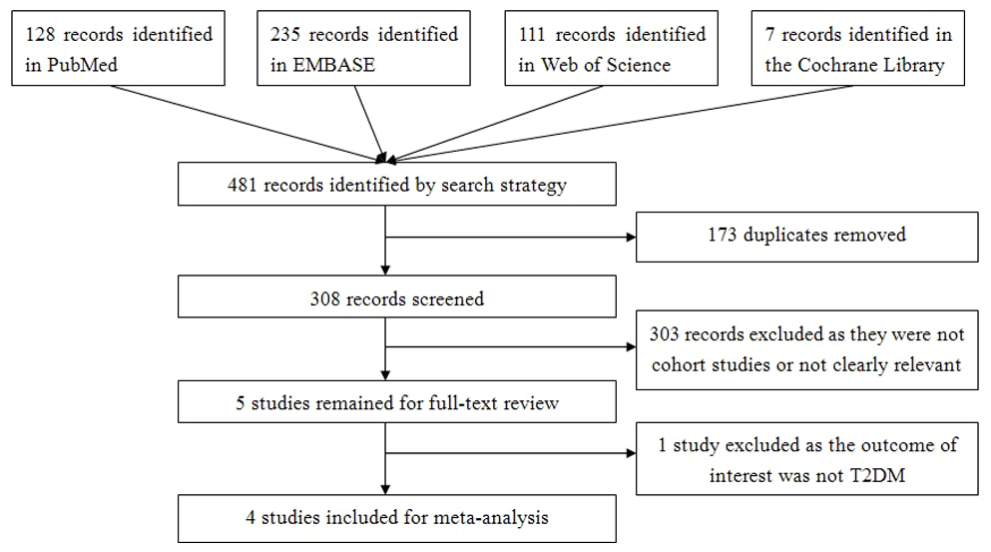
Flow chart of the selection of publications included in the meta-analysis.

Figure 1 shows the study selection process. A total of 481 articles were identified by the search strategy. 173 articles were removed as they were duplicates, left 308 articles for screening. By screening of titles or abstracts, 303 articles were excluded as they were not cohort studies or not clearly relevant. After reading the full text of the remained 5 articles, we excluded 1 articles [19] because the outcome of interest was not T2DM.. Finally, 4 studies [7], [20]–[22] were included for meta-analysis.

### Study characteristics

Table 2 summaries the main characteristics of the selected studies for analysis. The 4 studies were conducted in United States, Japan, Germany and South Korea, respectively. The population size per study ranged from 885 to 100,526, with a total of 112,351 participants involved. 3 studies consisted of males and females while 1 studies involved only females. Adjusted RRs (ORs/HRs) were reported in all studies. Adjustment for potential confounding factors differed across studies, and the main adjusted factors were age, sex, body mass index (BMI), alcohol drinking, family or parental history of diabetes, physical activity and hypertension. The maximum follow-up years were 4, 6, 7 and 24 years, respectively. Based on the quality assessment of NOS, 1 study was in high quality (scored 7) while the other 3 were in moderate quality (2 studies scored 6 and 1 study scored 5).

**Table 2:** Characteristics of studies included in the meta-analysis.

Study	No of diabetes/Sample size	Age (%Female)	Maximum follow-up year	Risk estimates (95%CI)	Define of exposed groups	Outcome assessment	Adjusted confounding factors	Quality score
Hayashino Y, 2008, Japan [7]	229/6,498	19–69(21)	4	HR1.81(1.06,3.19)	Those currently exposed to passive smoke but did not actively smoke, irrespective of past smoke	Fasting blood glucose level≧126 mg/dl,or random plasma glucose level≦200 mg/dl, or treatment with hypoglycemic medication, or self-reported history of diabetes.	Age, sex, BMI, physical activity, alcohol, family history of diabetes, hypertension, health promotion intervention, intake of sweetened beverage and vegetables, do not care about eating too much fat at all	5
Bernd K, 2010, Germany [21]	93/885	55–74(44)	7	OR2.40(0.996,5.90)	Never smokers exposed to passive smoke	Reported a physician diagnosis of T2DM or use of anti-diabetic medications, defined according to 1999 WHO criteria	Age, sex, parental history of diabetes, socioeconomic status, alcohol, physical activity, waist circumference, blood pressure, hypertriglyceridemia, log insulin, log adiponectin, HDL-cholesterol, intake of meat, sausage, salad, coffee, whole-grain bread and vegetables	6
Ko KP, 2011, South Korea [20]	465/4,442	40–69(84)	6	HR1.41(1.16,1.70)	Never smokers exposed to passive smoke	Fasting serum glucose level≧126 mg/dl, or serum glucose level after 2-hour OGTT≧200 mg/dl, or self-reported treatment with a hypoglycemic medication.	Age, sex, residential area, alcohol, education, waist circumference, regular exercise, hypertension history, total cholesterol, HOMA-IR, baseline glucose tolerance status	6
Zhang L, 2011, USA [22]	5,392/100,526	41–55(100)	24	RR1.16(1.00,1.35)	Never smokers regularly exposed to passive smoke	Reported a physician diagnosis of T2DM, or self-reported diabetes and ascertained by questionnaire	Age, race, BMI, physical activity, husband's education, alcohol, family history of diabetes, intake of total energy, magnesium, calcium, vitamin D, total trans fat, fiber from cereal, caffeine, total fat and saturated fat	7

### Main analysis

**Figure 2:**
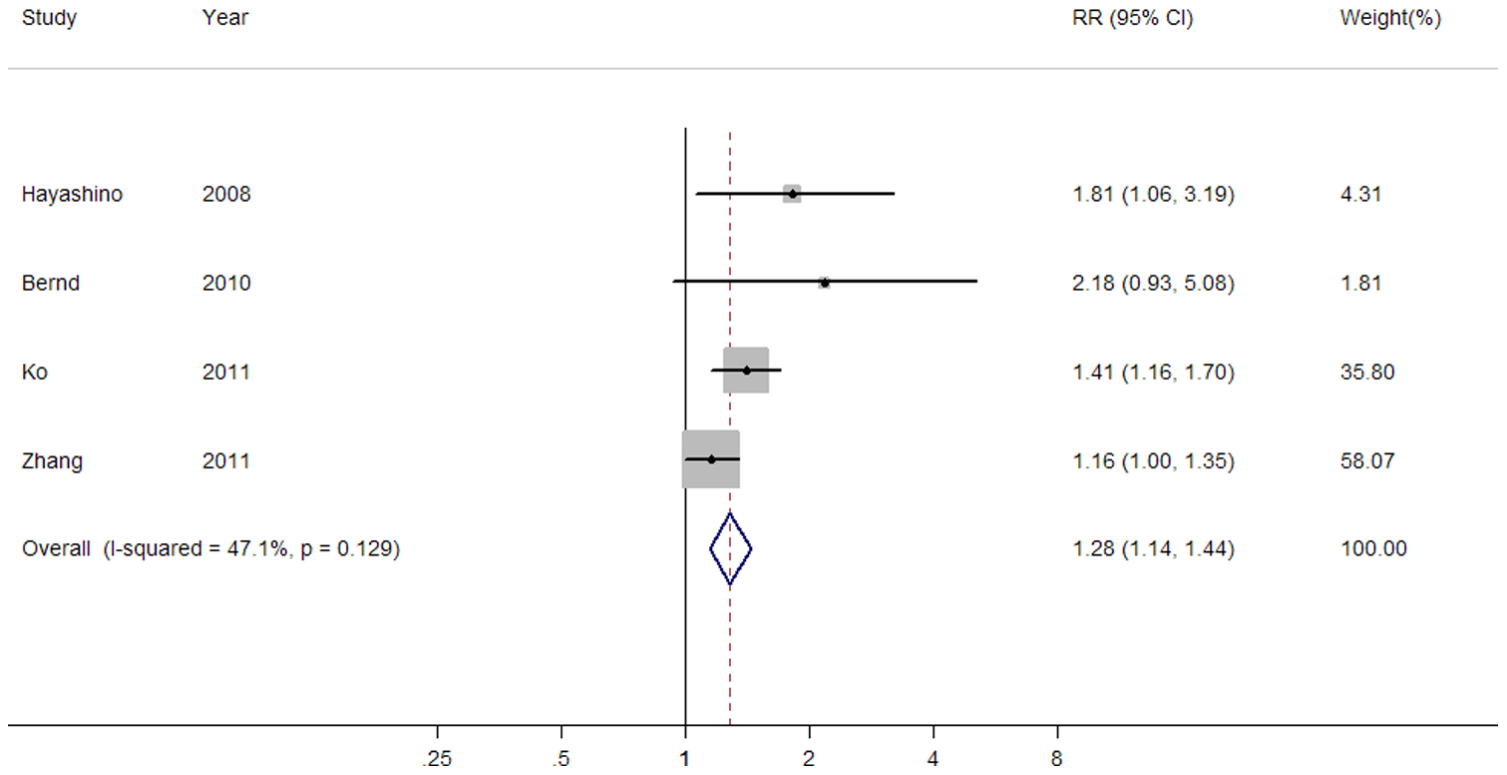
A forest plot of the association between passive smoking and type 2 diabetes risk.

Figure 2 shows a forest plot presenting the association between passive smoking and type 2 diabetes risk. No statistically significant heterogeneity across studies was found (*P*heterogeneity = 0.13, *I^2^* = 47.1%). Meta-analysis of the 4 included studies using fixed-effect model suggested an increased risk of T2DM in those who were exposed to passive smoke compared to those who were not (Overall RR = 1.28, 95% CI: 1.14–1.44).

### Subgroup and sensitivity analysis

Table 3 presents the results of subgroup analysis with both models according to study location, percentage of female, quality scores and adjustment for important confounding factors including BMI, family/parental history of diabetes and physical activity. For the results using fixed-model effect model, the associations between passive smoking and T2DM were similar to the overall result in subgroups. For the results by random-effect model, the pooled RRs were generally similar to the overall RR.

**Table 3:** Summary of the results of the association between passive smoking and type 2 diabetes.

Factor	Level	No. of studies	%I^2^(P value)	RR(95%CI)	
				Random effects model	Fixed effects model
Total		4	47.1(0.13)	1.36(1.11,1.65)	1.28(1.14,1.44)
Location	Asia	2	0.0(0.40)	1.45(1.21,1.73)	1.45(1.21,1.73)
	USA/Europe	2	51.4(0.15)	1.38(0.79,2.39)	1.18(1.02,1.37)
%Female	>50	2	59.7(0.12)	1.27(1.05,1.53)	1.25(1.11,1.41)
	≦50	2	0.0(0.72)	1.91(1.20,3.04)	1.91(1.20,3.04)
Study quality	High	1	0.0(0.00)	1.16(1.00,1.35)	1.16(1.00,1.35)
	Moderate	3	0.0(0.46)	1.47(1.24,1.76)	1.47(1.24,1.76)
Adjustment for confounding					
BMI	Yes	3	52.4(0.12)	1.45(0.98,2.15)	1.22(1.05,1.40)
	No	1	0.0(0.00)	1.41(1.16,1.71)	1.41(1.16,1.71)
Family/parental history of diabetes	Yes	3	52.4(0.12)	1.45(0.98,2.15)	1.22(1.05,1.40)
	No	1	0.0(0.00)	1.41(1.16,1.71)	1.41(1.16,1.71)
Physical activity	Yes	2	57.1(0.13)	1.33(0.89,2.00)	1.20(1.03,1.38)
	No	2	0.0(0.33)	1.44(1.20,1.74)	1.44(1.20,1.74)

Sensitivity analysis was conducted by omitting one study each time and re-calculating the pooled results. The overall risk estimates did not vary materially with a range from 1.21(95% CI: 1.05–1.40) to 1.47(95% CI: 1.24–1.76), indicating that the pooled RR was not substantially influenced by any of the individual study.

### Publication bias

Publication bias was not found in the included studies, as suggested by the Begg's and Egger's test (*P* = 0.17 by Egger's test and *P* = 0.16 by Begg's test). In addition, considering the limited number of included studies, we used the “fill and trim” method to assess the possible effects of potential publication bias on the pooled RR. Although The “fill and trim” method identified hypothetical 3 “missing” studies, the recalculated overall result with random-effect model continued to show a positive association between passive smoking and T2DM (RR = 1.27, 95% CI: 1.04–1.55).

## Discussion

To our knowledge, this is the first meta-analysis of prospective cohort studies on passive smoking and risk of T2DM. Result of the present analysis suggested that passive smoking was associated with a significantly increased risk of T2DM. Subgroup analysis, sensitivity analysis and publication bias test suggested the overall result of this analysis was robust.

A recent meta-analysis [23] based on 25 cohort studies found that active smoking was associated with a 44% increase in RR (RR = 1.44, 95%CI: 1.31–1.58) of T2DM. Nevertheless, in our analysis, passive smoking was associated with a 28% increase in RR of T2DM (RR = 1.28, 95%CI: 1.14–1.44), a smaller increase in RR than active smoking. Similar results were found in the study by Zhang L et al [22]. They found that active smoking can increase 39% (≤14 cigarettes per day, RR = 1.39, 95%CI: 1.17–1.64) or 98% (≥25 cigarettes per day, RR = 1.98, 95%CI: 1.57–2.36) risk of T2DM among nonsmokers, while passive smoking was only associated with 10% (occasionally exposure, RR = 1.10, 95%CI: 0.94–1.23) or 16% (regularly exposure, RR = 1.16, 95%CI: 1.00–1.35) increase in risk of T2DM for nonsmokers who were not exposed to passive smoking. Similar results were also reported in the studies on coronary heart disease and stroke [24], [25]. These might be explained by the dilution of passive smoke by room air, which makes a non-smoker be exposed to less tobacco smoke than an active smoker [26].

Although passive smoking was associated with a smaller increase in RR of diabetes compared with active smoking, the burden of diabetes caused by the high prevalence of passive smoking in workplace and at home should not be neglected. One smoker might result in the exposure of passive smoke to several workmates or families, especially in those countries with insufficient anti-smoking legislation. In places where people are not allowed to smoke, the concentration of nicotine in the air is lower than in places where smoking is allowed [27]. This implies the importance of anti-smoking legislation to the reduction of T2DM risk by population level. Moreover, to achieve a significant reduction of T2DM in the burden to society, prohibitions on both passive and active smoking should be targeted.

Several mechanisms might be involved in the effect of passive smoking on diabetes. The environmental tobacco smoke consists of nearly 5,000 chemical compounds, including 43 known carcinogens, carbon monoxide, nicotine and other toxic ingredients [28]. Nicotine is an important ingredient in cigarette smoke that can cause insulin resistance by affecting insulin action [29]. Animal studies also suggested that prenatal or neonatal exposure to nicotine will lead to loss of pancreatic β-cells [30]. In addition, epidemiological studies have found that exposure to environmental tobacco smoke in the childhood was associated with increased risk of pancreatic cancer [31], [32]; this suggests that tobacco smoke might have a chronic toxic effect to the pancreas. Third, like active smoking, passive smoking has been related to oxidative stress, systemic inflammation and endothelial dysfunction [33], which were strongly involved in insulin resistance and diabetes risk [22].

There are several potential limitations in this study that warrant consideration. First, the results of this analysis were based on prospective cohort studies. However, observational studies cannot prove causality.

Second, although all the included studies controlled several known risk factors for T2DM, including age, sex, BMI, alcohol, and physical activity etc, residual or unmeasured confounding may still affect the observed association. Furthermore, there was heterogeneity between studies in regard to adjusting for confounding factors, which may lead to misleading overall results.

Third, as the exposure status of passive smoking was generally self-reported, there was a possibility that participants tend to narrow their exposure status; and this may lead to underestimate or exaggerate of the risk estimates if the lessened extent in two groups were different. However, Sasaki et al. in their study found that self-reported passive smoking might lead to underestimate the true exposure status and thus underestimate the strength of association between passive smoking and T2DM risk [34].

Fourth, although no publication bias was found by the Begg's and Egger's test, the power to detect bias of this two tests was low with small numbers of studies [16], [17]. However, although the risk estimates were slightly reduced, the fill and trim analysis showed no significant change of the general result. Nevertheless, the possibility of publication bias cannot be fully excluded by this analysis.

Finally, our analysis failed to assess a dose-response relationship between passive smoking and T2DM, because there was only one included study that explored this relationship, although a dose-response relationship was found both in workplace and home in this study [20].

In conclusion, the present meta-analysis suggests that passive smoking is associated with an increased risk of T2DM. However, considering the limited number and moderate quality of included studies, further well-designed studies are warranted to confirm this observed association. The dose-response relationship should be well explored by studies in the future.

## Supporting Information

Table S1 **PRISMA 2009 Checklist.**
(DOC)
